# Deciphering the Immune Microenvironment at the Forefront of Tumor Aggressiveness by Constructing a Regulatory Network with Single-Cell and Spatial Transcriptomic Data

**DOI:** 10.3390/genes15010100

**Published:** 2024-01-15

**Authors:** Kun Xu, Dongshuo Yu, Siwen Zhang, Lanming Chen, Zhenhao Liu, Lu Xie

**Affiliations:** 1 College of Food Science and Technology, Shanghai Ocean University, Shanghai 201306, China; xukun01102021@126.com; 2 Shanghai-MOST Key Laboratory of Health and Disease Genomics, The Institute of Genome and Bioinformatics, Shanghai Institute for Biomedical and Pharmaceutical Technologies, Shanghai 200037, China; yds290zcf@163.com (D.Y.); zhangsiwen07@163.com (S.Z.); 3 Key Laboratory of Quality and Safety Risk Assessment for Aquatic Products on Storage and Preservation (Shanghai), China Ministry of Agriculture, College of Food Science and Technology, Shanghai Ocean University, Shanghai 201306, China; lmchen@shou.edu.cn

**Keywords:** single-cell RNA sequencing, spatial transcriptomics, intercellular gene regulatory network construction, frontier of aggression, ER-positive breast cancer

## Abstract

The heterogeneity and intricate cellular architecture of complex cellular ecosystems play a crucial role in the progression and therapeutic response of cancer. Understanding the regulatory relationships of malignant cells at the invasive front of the tumor microenvironment (TME) is important to explore the heterogeneity of the TME and its role in disease progression. In this study, we inferred malignant cells at the invasion front by analyzing single-cell RNA sequencing (scRNA-seq) and spatial transcriptomics (ST) data of ER-positive (ER+) breast cancer patients. In addition, we developed a software pipeline for constructing intercellular gene regulatory networks (IGRNs), which help to reduce errors generated by single-cell communication analysis and increase the confidence of selected cell communication signals. Based on the constructed IGRN between malignant cells at the invasive front of the TME and the immune cells of ER+ breast cancer patients, we found that a high expression of the transcription factors FOXA1 and EZH2 played a key role in driving tumor progression. Meanwhile, elevated levels of their downstream target genes (ESR1 and CDKN1A) were associated with poor prognosis of breast cancer patients. This study demonstrates a bioinformatics workflow of combining scRNA-seq and ST data; in addition, the study provides the software pipelines for constructing IGRNs automatically (cIGRN). This strategy will help decipher cancer progression by revealing bidirectional signaling between invasive frontline malignant tumor cells and immune cells, and the selected signaling molecules in the regulatory network may serve as biomarkers for mechanism studies or therapeutic targets.

## 1. Introduction

Breast cancer is the most common gynecological cancer worldwide, and it ranks second in female cancer deaths [1]. Although breast cancer mortality has declined by 42% in the past three decades, breast cancer remains the second leading cause of cancer mortality in women [2]. Of these, ER+ breast cancer is the most common subtype of breast cancer, accounting for approximately 75% of cases. Even after five years of adjuvant endocrine therapy, women with ER-positive, early-stage breast cancer still had a persistent risk of recurrence and death from breast cancer for at least 20 years after the original diagnosis [3]. Breast cancers have diverse cellular microenvironments, wherein heterotypic interactions are important in defining disease etiology and response to treatment [4]. Invasive metastasis is a critical determinant of prognosis in malignant tumors. The parenchymal cells within tumors exhibit significant heterogeneity, displaying diverse biological characteristics. However, the aggressive tumor cells located at the leading edge of the tumor often play a crucial role in invasion and metastasis. Consequently, there is an urgent need to comprehend the signaling interactions between malignant cells and the immune cell populations present at the forefront of invasiveness within the tumor microenvironments (TMEs) of ER+ breast cancer.

With the boom in sequencing technology, single-cell RNA sequencing (scRNA-seq) has become the state-of-the-art approach for unraveling the heterogeneity and complexity of RNA transcripts within individual cells, as well as revealing the composition of different cell types and functions within highly organized tissues/organs/organisms [5]. In our previous work, we have proved that the construction of the gene regulatory network improves the accuracy and integrity of deciphering signaling molecules in intercellular communication [6]. However, the precise spatial localization of cells within the TME and how the TME influences their interactions remain elusive. Recent advances in the spatial transcriptome (ST) have enabled the location of dozens of different cells in the TME to be determined simultaneously. This is essential for understanding tumor–stromal crosstalk. Therefore, combining single-cell transcriptomic data with other high dimensional ST data will help us to resolve the spatial distribution of cells in a more comprehensive manner [7]. In addition, the construction of intercellular gene regulatory networks (IGRNs) facilitates a deeper understanding of the interactions between different cell types in the TME, revealing potential associations and regulations, thus revealing the fine regulatory mechanism, and which cells or which signaling molecules play roles in cancer invasion and progression.

In our previous works, we have demonstrated the important roles played by plasma cells [8] and macrophages [6] in the TME for cancer stage development from more mild to more advanced. In this study, we continue to focus on macrophages, plus other potential immune cells, such as the well-investigated T cells, in solid tumors. Recently, multiple studies reported that the TME is overwhelmingly dictated by macrophages in tumors including breast cancers, influencing pivotal processes such as angiogenesis, extracellular matrix reconfiguration, cellular proliferation, metastasis, and immunosuppression [9,10,11,12]. The interaction between tumor and immune cells contributes to a deeper understanding of the mechanisms of cancer initiation and progression. More optimally, such interaction should be studied in the TME at the frontiers of cancer aggressiveness, and such goals would require spatial omics data.

In this study, we tried to combine scRNA-seq and ST data of ER+ breast cancer tissues, in hopes to demonstrate a workflow of dissecting the events occurring at the forefront of tumor infiltration. We further refined and updated our previously reported IGRN construction method [6] and provided a pipeline on GitHub (cIGRN), containing two methods for constructing gene regulatory networks (https://github.com/xukun01102021/cIGRN, accessed on 9 December 2023). By applying cIGRN on the integrated scRNA-seq and ST data of ER+ breast cancer patients, we described the signaling molecules between macrophages/T cells and malignant cells at the invasion front of the spatial breast cancer tissue.

## 2. Materials and Methods

### 2.1. Single-Cell Transcriptome Data Collection and Preprocessing

To characterize the composition and functional status of ER+ breast cancers, we collected several sets of single-cell transcriptome data from public databases. The first dataset, GSE161529 [13], provided single-cell transcriptome data from 13 cases of normal breast tissue. The second dataset, GSE176078 [14], provided single-cell transcriptome data from 11 cases of ER+ breast cancer (referred to as ER+ tumor). Both datasets were generated using 10x Genomics (Pleasanton, CA, USA) sequencing technology. For a more accurate analysis, we applied quality measures to the original gene–cell barcode matrix for each cell based on the following parameters: mitochondrial genes (≤20%), unique molecular identifiers (UMI) (from 200 to 60,000), and gene counts (>200). After quality control, 58,058 cells from normal breast tissue (Normal) and 38,121 cells from ER+ breast tissue (ER+ tumor) were retained for further analysis.

To validate the regulatory network changes associated with the aggressive front of ER+ breast cancer, we retrieved a third set of single-cell transcriptomic data GSE164898 [15], which was used to provide normal breast samples. We also retrieved the GSE161529 dataset [13], which was used to provide tissue samples from ER+ tumor samples. In the test set, we used normal breast tissue samples provided by the GSE161529 dataset, while in the validation set, we used the GSE161529 dataset under the same GSE number, which provided ER+ tumor samples. After quality control, a total of 69,405 cells were selected for subsequent analysis (including 18,688 and 50,717 cells from normal breast samples and ER+ tumor sample tissue, respectively). The same analysis strategy was used for the gene regulatory networks in the validation dataset.

### 2.2. Dimension Reduction and Clustering Analysis of Single-Cell Transcriptome Data

We employed the R package Seurat (version 4.3.0) [16] to normalize and scale the scRNA-seq data using the “NormalizeData” and “ScaleData” functions. The top 2000 highly variable genes were identified using the “FindVariableFeatures” function. A principal component analysis (PCA) was performed on these variable genes, and batch effects were mitigated using the Harmony algorithm (version 0.1.0) [17]. The ElbowPlot function within the Seurat R package was utilized to rank principal components, where this process involves randomly permuting subsets of data and calculating PCA projection scores. Upon reaching a point of inflection at the 25th principal component, the first 25 principal components were used for a Uniform Manifold Approximation and Projection (UMAP) analysis using the “RunUMAP”. Moreover, the “FindAllMarkers” function was employed for the identification of gene expression markers. Subsequently, cell types within this study were annotated utilizing the R package SingleR (version 1.8.1) [18], the CellMarker dataset [19], and cell-specific marker genes.

### 2.3. Combining Spatial Transcriptome (ST) Data with scRNA-seq Data

To elucidate spatial cellular localization, we collected two datasets from human ER+ breast cancers (BRCA1, BRCA2) from the 10x Genomics (Pleasanton, CA, USA) database. The Visium Spatial platform from 10x Genomics (Pleasanton, CA, USA) employs spatial barcoding via a default scheme of mRNA-binding oligonucleotides to capture gene expression data from ST slides. Space Ranger v1.2 and Space Ranger v1.3 were used for quality checking and raw sequencing read mapping of BRCA1 and BRCA2 ST data, respectively. Gene speckle matrices derived from the processing of ST data from Visium samples were subjected to analysis using the Seurat package in R (version 4.1.2). Normalization was conducted via the SCTransform function, sorting genes by residual variance, and selecting 3000 features as variables. The initial 30 principal components underwent condensation and clustering via principal component analysis (PCA). To augment cell-wise expression information to the ST, we integrated previously acquired single-cell transcriptome data from 11 ER+ breast cancer patients (GSE176078) [14]. After downscaling the clusters, the annotated scRNA-seq was normalized using the SCTransform function. Subsequently, we computed the spatial distribution of each cell type in the scRNA sequencing dataset, estimating the quantity of each cell type at each spatial point in the transcriptome using Seurat (version 4.3.0). Spatial feature expression plots were generated using Seurat’s SpatialFeaturePlot function.

Moreover, to avoid errors caused by a single tool, we further applied CellTrek (v0.0.94) [20] to facilitate the visualization of cellular spatial localization. Initially, the traint function was utilized to co-embed ST and annotated scRNA-seq datasets. Subsequently, the CellTrek function mapped individual cells to their spatial coordinates. Finally, the CellTrek results were interactively visualized using celltrek_vis. Additionally, to be more precise and quantitative, the kdist function in CellTrek was used to calculate the K-distance between the query cell and the reference cell based on spatial coordinates; therefore, we could infer the distances between malignant cells and other cell populations.

### 2.4. Identification of Malignant Cells from scRNA-seq Data of ER+ Breast Cancer

In order to discern between malignant cells within the epithelial cells, we assessed the somatic large-scale chromosome copy number variation (CNV) score for individual epithelial cells utilizing the infercnv R software package (version 1.1.1). Compliant with data prerequisites outlined in the project’s GitHub repository (https://github.com/broadinstitute/inferCNV, accessed on 2 January 2023), a raw counts matrix, annotation file, and gene/chromosome position file were meticulously prepared. In this analytical process, normal epithelial cells (referred to as “normal epi”) were designated as the reference standard for normality, the ER+ tumor tissue was used as the observation group. Employing default parameters (cut.off = 0.1; cluster_by_groups = T), the CNV score was computed as the cumulative value of the CNV region. Using the copy number score of normal epithelial cells as a reference, cell populations with CNV, especially those with significantly higher copy number scores than normal cells, were considered malignant.

### 2.5. Developmental Trajectory of Malignant Epithelial Cells

Monocle 2 (version 2.18.0) was employed for the inference of cell trajectories in malignant epithelial cells, presuming that a one-dimensional “time” could encapsulate the higher-dimensional expression values, a paradigm commonly referred to as single-cell pseudotime analysis [21]. A systematically standardized pipeline was enacted, encompassing logarithmic normalization and dimensionality reduction facilitated by DDRTree. This was succeeded by the visualization of cell trajectories and their respective positions, portrayed within a two-dimensional layout resembling a branching tree structure.

### 2.6. Immune Cell Infiltration Evaluation and Survival Analysis

To gain a more precise comprehension of immune cell infiltration in ER+ breast cancer and its correlation with patient survival, we acquired breast cancer (BRCA) mRNA expression data and pertinent clinical information from the TCGA database via the UCSC Xena platform (http://xena.ucsc.edu/, accessed on 2 November 2022). Subsequently, ER+ breast cancer-specific data were isolated using PAM50Call_RNAseq (luminal A/B) from Phenotypic Information. CIBERSORT was performed to estimate the infiltrated proportion of 22 immune cell types in TCGA BRCA [22]. The patients were divided into two groups (high-risk and low-risk groups) according to the proportion of infiltrated immune cells. Then, the relationship with survival was evaluated using the R package survival (version 3.3.1) [23]. The “surv_cutpoint” algorithm within the R package survminer (version 0.4.9) was utilized to compute optimal thresholds, and this approach was employed for all survival analyses. To retain a broader range of variables in the multivariate analysis, a lenient criterion was adopted, considering variables with *p*-values < 0.05 as significantly associated with prognosis.

To corroborate the impact of signaling molecules within the regulatory network on the prognosis of ER+ breast cancer patients, we employed the GEPIA2 [24] tool to assess the association between target genes within the gene regulatory network and the survival period of breast cancer patients.

### 2.7. Tool Development for the Construction of Intercellular Gene Regulatory Network (cIGRN)

Aiming to investigate the role of immune cells in tumor development, we constructed a cell-interaction-based IGRN between malignant and immune cells in the TME. First, we constructed a preliminary version of the cell-interaction gene regulatory network using scMLnet (version 0.1.0) [25], with the following parameters “pval” = 0.05, “logfc” = 0.15. The initial version of the cell-interacting gene regulatory network included ligand–receptor (L–R), receptor–transcription factor (R–TF), and transcription factor–target gene (TF–target) information. pySCENIC (version 0.11.2) [26] was then used to identify transcription factors in malignant cells and to prune the gene regulatory network. Finally, differentially expressed genes (DEGs) were screened using the Seurat “Findmarker” function to identify target genes regulated by transcription factors in the gene regulatory network. In this study, the pruned gene regulatory network was used as the final version of the cell interaction-based microenvironmental gene regulatory network for subsequent analysis. The IGRN strategy was published by our group [6]. In this work, we have updated it and embedded two methods for constructing gene regulatory networks into one pipeline (cIGRN). The building code for the network can be obtained from this site (xukun01102021/cIGRN (github.com), accessed on 9 December 2023).

### 2.8. Statistical Analysis and Functional Enrichment Analysis

A functional enrichment analysis was performed using clusterProfiler (version 4.2.2) in R. Genes were annotated with different functions using gene set enrichment analysis (GSEA). Gene sets with *p*.adjust < 0.05 in the statistical analysis were thought of as significantly enriched from the MSigDB HALLMARK set. All the statistical analyses in this study were calculated in R (version 4.1.2). The data visualization was performed using correspondence functions in the R packages used for this study or using ggplot2 (version 3.4.1) in R.

## 3. Results

### 3.1. Single-Cell Transcriptome Profiles of ER+ Breast Cancer

**Figure 1:**
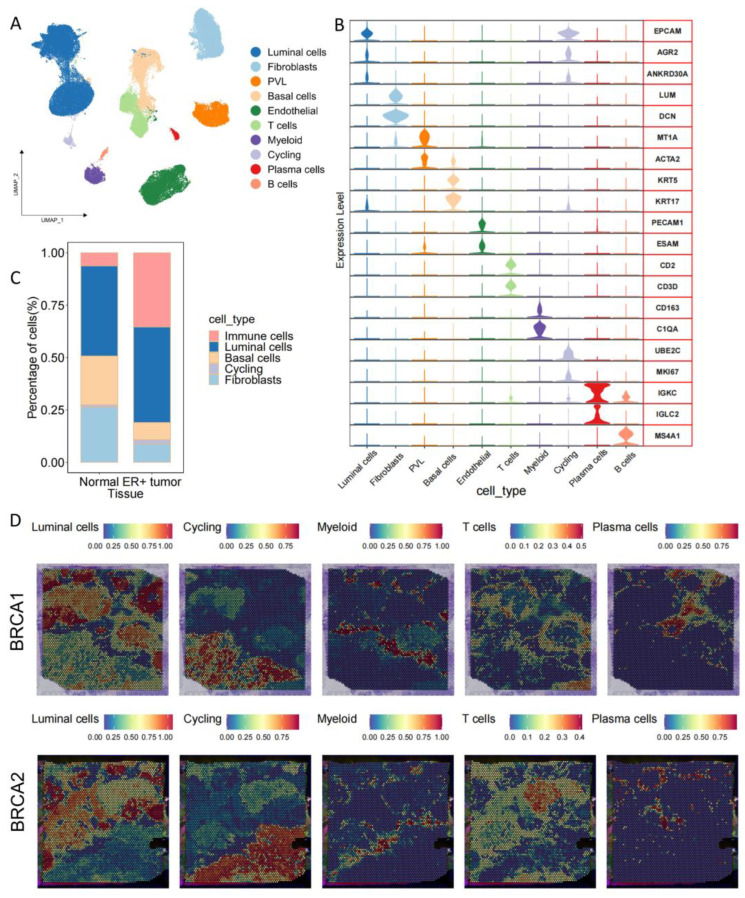
Single-cell and spatial transcriptome (ST) profiles of human normal and ER+ breast cancer tissues. (**A**) UMAP plot for showing different cell type populations. (**B**) Overlay violin plot showing expression markers for ten cell types. (**C**) Proportion of cells in normal and ER+ tumor, immune cells (myeloid cells, T cells, B cells, plasma cells). (**D**) The unbiased clustering of ST points shows the spatial location of the main cell types, with red representing high expression of this group at that location and blue representing low expression.

To comprehensively characterize the single-cell landscape of ER+ breast cancer, the scRNA-seq data of 96,179 cells from 13 normal breast samples and 11 ER+ breast cancer patient samples (Table S1) were integrated for analysis. Cell clusters were assigned based on the expression levels of marker genes (Figure 1A,B). Ten main cell types were annotated in TME: four types of immune cells (including T cells, myeloid cells, B cells, and plasma cells), three types of epithelial cells (including luminal cells, basal cells, and cycling cells), and three types of stromal cells (including fibroblasts, endothelial cells, and PVL cells). Upon integration, the cells primarily clustered based on the dataset origin (Figure S1A). Notably, a higher proportion of epithelial cells (luminal and cycling cells) was observed in ER+ tumors as the disease progressed (Figure 1C). In addition, to elucidate the tumor environment of ER+ breast cancer, we estimated the spatial distribution of cells using Seurat and CellTrek, while inferring the spatial distance between cells (Figure 1D and Figure S1B–D). The results showed that immune cells were enriched around epithelial cells (luminal cells and cycling cells), suggesting a physical interaction between epithelial cells and immune cells. This observation suggests that epithelial cell proliferation in the TME may be closely related to immune cell interactions.

### 3.2. Inferring Malignant Cells at the Frontiers of Invasiveness

Breast cancer originates from mammary epithelial cells [27] and breast cancer is mainly driven by DNA copy number changes [28]. Generally speaking, CNV is an important component of SV (Structural Variation); it is the duplication or deletion of an entire gene segment on the genome, caused by genome rearrangement, and the area involved is much larger than the SNV (single nucleotide variants) of a single point mutation. Therefore, CNV is considered a fundamental feature of a mutated genome and is taken into single-cell data analysis to be used to identify malignant cells from normal ones. The R package infercnv was used to identify non-malignant cells and malignant cells by analyzing aneuploidy and chromosomal CNV [29]. Taking the expression profile of normal breast tissue as a reference and an ER+ tumor as the observation group (Figure S2), we found that the copy number of cells provided by the ER+ tumor tissue in epithelial cells (luminal cells, cycling cells, and basal cells) showed significant changes and these cell types showed increased expression levels of tumor markers associated with ER+ breast cancer. Furthermore, a strong correlation was observed between luminal cells, basal cells, and cycling cells (Spearman correlation coefficient > 0.80). Based on these findings, we hypothesized that epithelial cells represent malignant cells in the context of ER+ breast cancer.

**Figure 2:**
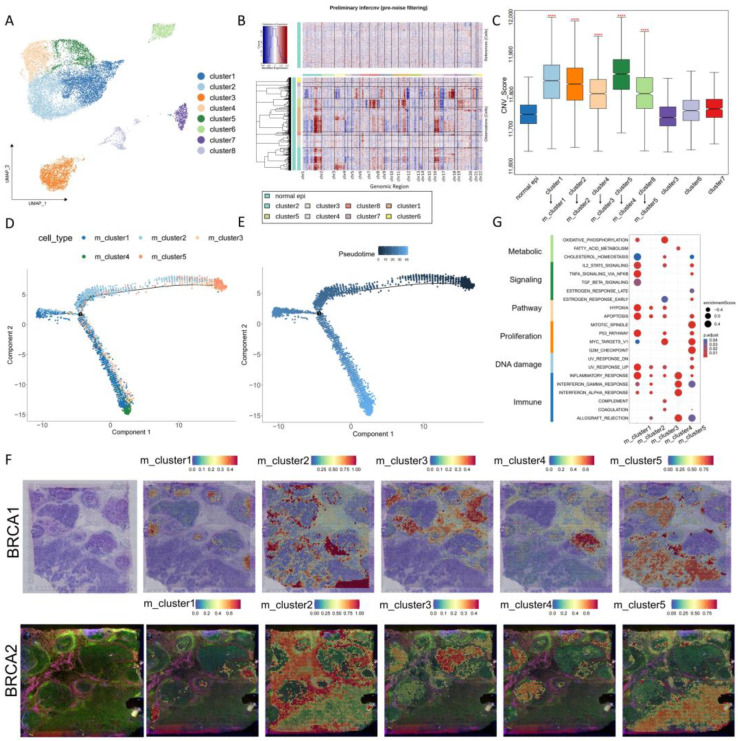
Identification of malignant epithelial cells in ER+ breast cancer and their developmental trajectory. (**A**) A single-cell atlas of epithelial cells. (**B**) Heatmap illustrating extensive CNV profiles across epithelial cell clusters. Red and blue shades denote elevated and reduced CNV levels, respectively, with reference to normal epithelial cells. (**C**) Box plot comparing CNV scores across diverse epithelial cell clusters (“****” means *p*-value < 0.0001). (**D**) Pseudotemporal exploration of cell trajectories in malignant cells featuring genes of high variability. (**E**) Evolutionary trajectory of malignant cells. (**F**) Spatial representation of malignant cell cluster locations within the tissue image. (**G**) The bubble plot demonstrates the functional enrichment analysis of malignant cell subpopulations, where darker red represents stronger correlation.

In the spatial samples of the TME, malignant cells exhibit inherent heterogeneity, generating unique spatial niches that collectively drive tumor progression. Of particular note, the aggressive tumor front plays a key role in the invasion and metastasis of cancer [30]. To infer malignant cells at the invasive front, the isolation of epithelial cells from the ER+ tumor was performed for subgroup analysis. Eight distinct cell clusters were identified (Figure 2A). Using the expression profiles of normal epithelial tissues as a reference, we found that clusters 1, 2, 4, 5, and 8 were found to exhibit distinct patterns of chromosomal copy number variation (Figure 2B), and their CNV scores were significantly higher than those of the reference cell cluster (Figure 2C) (*p*-value < 0.05). Thus, they were classified as malignant cells (m_cluster1, m_cluster2, m_cluster3, m_cluster4, and m_cluster5), in the same order as primitive cells, respectively. The other three clusters were designated as normal epithelial cells (n_cluster1, n_cluster2, and n_cluster3) in the ER+ tumor. Furthermore, we inferred the evolutionary dynamics of malignant cells (Figure 2D,E): m_cluster2, m_cluster3, and m_cluster5 are located at the beginning of the developmental trajectory, indicating their strong invasion and proliferation capabilities. Notably, spatial location mapping indicated that m_cluster2, m_cluster3, and m_cluster5 were located at the leading edge of the tumor tissue (Figure 2F). Therefore, the cluster of cells containing m_cluster2, m_cluster3, and m_cluster5 in cell track state 3 was inferred to be composed of malignant cells at the invasive front (Figure S3A,B), with m_cluster2 being the dominating subgroup.

We noticed distinct variations in the ratio of malignant cells among 11 ER+ tumor samples (Figure S3C). Furthermore, these malignant cells displayed different spatial distributions in two cases of ER+ tumor tissues with ST data (Figure 2F), emphasizing the heterogeneity of malignant cells in ER+ breast cancers across various patients. We also identified functional disparities among these malignant cells (Figure 2G). Specifically, the m_cluster2 exhibited enrichment in pathways associated with cell cycle regulation and metabolism, such as MYC and Oxidative Phosphorylation. This discovery indicates a potentially pivotal role played by the m_cluster2 cell population located at the invasive front in driving tumor progression. Additionally, the enrichment of multiple immune pathways, as well as the enrichment of immune cells around malignant cells, suggests intricate interactions between malignant cells and the immune system within the TME, particularly at the invasion front. This may underscore the heterogeneous nature of the immune microenvironment surrounding different malignant cell populations.

### 3.3. The Immune Microenvironment at the Invasive Front

**Figure 3:**
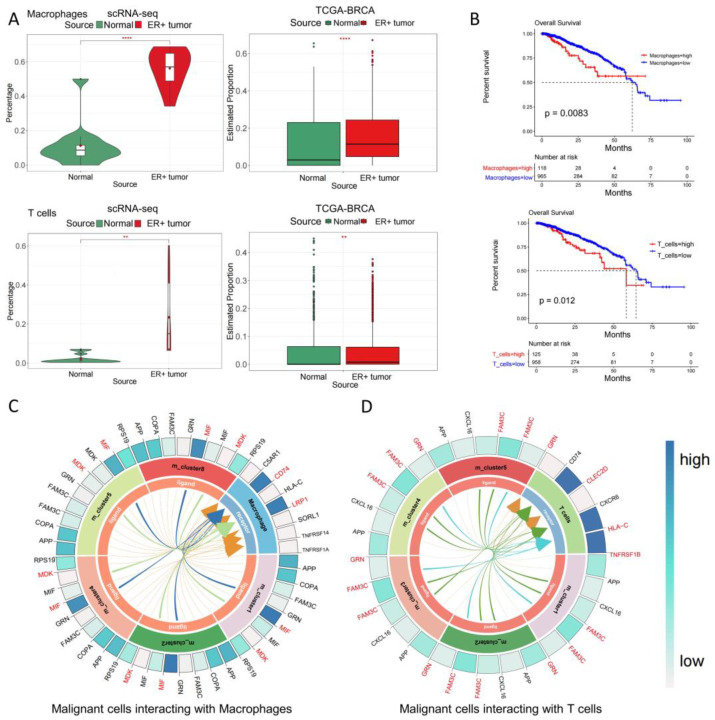
Analysis of proportional changes and survival of immune cells, and ligand–receptor interactions between malignant cells and macrophages/T cells. (**A**) Comparative statistical assessment of macrophage and T cell proportions within cells sourced from normal and tumor samples, based on both single-cell data and TCGA data (“**” means *p*-value < 0.01, “****” means *p*-value < 0.0001). (**B**) Survival analysis utilizing TCGA-BRCA dataset, focusing on T cells and macrophages. (**C**) The diagram depicts ligand–receptor interactions between malignant cells and macrophages, where darker hues in the outer circle indicate elevated weights. (**D**) The diagram illustrates ligand–receptor interactions between malignant cells and T cells, with intensified shades in the outer circle denoting augmented weights.

To understand the immune microenvironment of malignant cells at the invasive front and the key immune cell types that have the potential to promote malignant cell development, we first analyzed the enriched immune cell types (T cells, myeloid cells, and plasma cells) at the invasion front (Figure 1D and Figure 2F). Additionally, to further characterize specific subtypes of myeloid cells, we performed a subpopulation analysis of myeloid cells. Notably, a single-cell ST data analysis revealed that primitive myeloid cells were primarily macrophages (Figure S4A,B). Subsequently, through single-cell transcriptome data and TCGA-BRCA data, it was observed that higher proportions of T cells and macrophages were observed in ER+ breast cancer samples. Further, high proportions of T cells and macrophages in tumor samples were associated with poor prognosis in breast cancer patients (Figure 3A,B). However, the abundance of plasma cells did not change significantly between normal and tumor samples and was not significantly related to the prognosis of breast cancer patients (Figure S4C). Therefore, the proliferation, invasion, and metastasis of malignant cells may be promoted by interacting with T cells and macrophages. Next, we focused on the complex mechanisms between T cells/macrophages and malignant cells at the invasive front of ER+ cancer samples.

### 3.4. Ligand–Receptor Interactions between Malignant Cells and Macrophages/T Cells

Malignant cells possess sophisticated mechanisms to evade and suppress immune responses, to help with their invasion and progression. To decipher such mechanisms, it is important to identify the signaling molecules of immune cells mediated by malignant cells. To this end, we first conducted a comprehensive analysis of the ligand–receptor interactions between malignant cells and macrophages/T cells using the cellphoneDB database.

Intriguingly, we identified specific ligand–receptor interactions, namely MIF-CD74 and MDK-LRP1, between malignant cells and macrophages (Figure 3C), and ligand–receptor interactions for malignant cells and T cells, namely FAM3C-HLA-C/CLEC2D and GRN-TNFRSF1B (Figure 3D). Importantly, these interactions were observed across multiple tumor clusters, highlighting their potential relevance in various contexts. The MIF-CD74 axis can play an important regulatory role in innate immune system responses to cancer by inducing an immunosuppressive environment that supports tumor progression [31,32]. MDK-LRP1 is an essential factor for immunosuppressive macrophage differentiation [33]. In addition, it was reported that FAM3C can induce the epithelial-to-mesenchymal transition (EMT) process in cancer, which is critical for tumor invasion, metastasis, and recurrence [34]. The gene regulatory network helps with bestowing tumor cells with aggressive phenotypic features [35]. Overall, malignant cells continue to proliferate by creating an immunosuppressive environment through mediated macrophage/T cells.

### 3.5. Pipeline for the Construction of Intercellular Gene Regulatory Network

**Figure 4:**
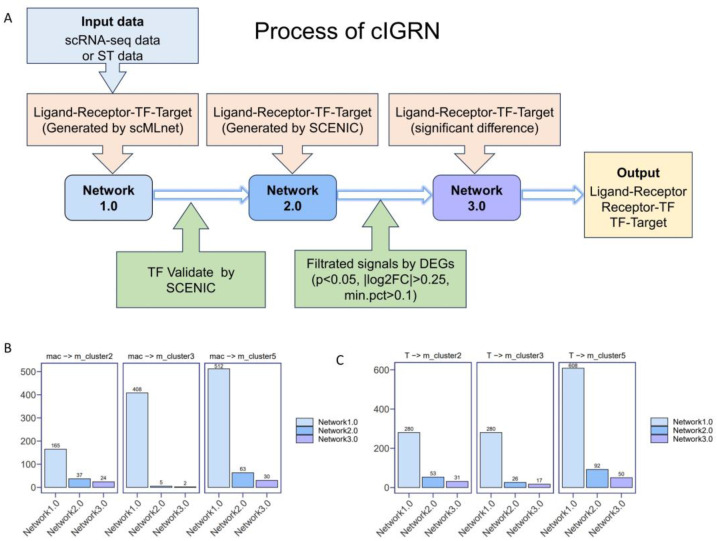
cIGRN process. (**A**) Flowchart for the construction of cIGRN. (**B**) Macrophages mediate the amount of signaling in the IGRN of invasive frontline malignant cells. (**C**) T cells mediate the amount of signaling in the IGRN of invasive frontline malignant cells.

To further understand whether immune cells at the invasion front regulate the proliferation of malignant cells, and to understand the downstream signaling molecules that regulate malignant cells, we devised a pioneering methodology for establishing the IGRN (including ligand–receptor–transcription factor (TF)–target gene). First, we constructed a preliminary version of the cell-interaction gene regulatory network using scMLnet. pySCENIC was then used to identify transcription factors in malignant cells and to prune the gene regulatory network. Lastly, a screen using differentially expressed genes (DEGs) was performed to identify target genes regulated by transcription factors in the gene regulatory network. The IGRN construction method aims to simplify the identification of signaling molecules and minimize false positive results by employing a unified network construction and screening pipeline (Figure 4A). For example, the signal is reduced from 165 to 24 for the IGRN mediation of m_cluster2 by macrophages (Figure 4B). The advantages of this process over our previous work [6] are the accuracy of screening for signaling molecules and the greater ease of application to the cellular communication analysis of ST data. The building code for the network can be obtained from this site (xukun01102021/cIGRN (github.com), accessed on 9 December 2023). In this study, we focused on constructing the IGRN between macrophages/T cells and malignant cells at the invasion front.

### 3.6. IGRN between Macrophages/T Cells and Malignant Cells at the Invasive Front

**Figure 5:**
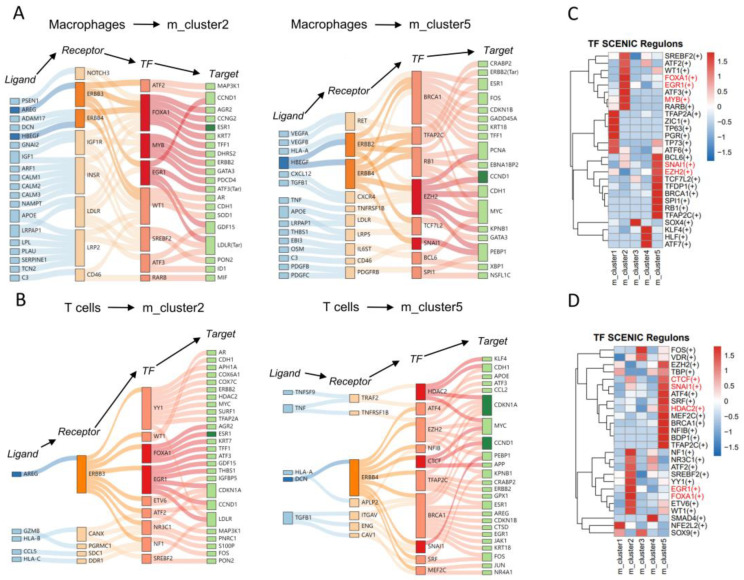
IGRN of macrophages/T cells regulating malignant cells. (**A**) Macrophage activities within the IGRN of m_cluster2 and m_cluster5. The network entails ligand–receptor pairs, TFs, and their respective targets. (**B**) T cell functionalities within the IGRN of m_cluster2 and m_cluster5. The network involves ligand–receptor interactions, TFs, and their associated targets. (**C**) Heatmap depicting enriched transcription factors (TFs) within the regulatory network of macrophages across distinct malignant cell clusters. (**D**) Heatmap illustrating enriched TF expression within the regulatory network of T cells across individual malignant cell clusters.

Our goal is to identify the signaling network initiated by immune cells to regulate malignant cell invasion and proliferation. By investigating the IGRN, we found that macrophages interacted with malignant cells in m_cluster2 by the AREG-ERBB3-FOXA1-ESR1 IGRN path (Figure 5A). The mediator target gene ESR1 is highly expressed in the tumor (Figure S5C). FOXA1 cooperates with Estrogen Receptor-1 (ESR1) in regulating the gene expression in ER-positive luminal breast cancer cells, as evidenced by a clinical data analysis indicating that high levels of FOXA1 improve the survival of ESR1^Low^ patients, but worsen the survival of ESR1^High^ patients of breast cancer [36]. In addition, macrophages act on the m_cluster5 to activate the TF–target gene: EZH2-CCND1 (Figure 5A). A high level of EZH2 is implicated in tumorigenesis and correlates with poor prognosis in various tumor types, including breast cancer, ovarian cancer, etc. [37,38]. Meanwhile, the EZH2-mediated target gene CCND1 was upregulated in the tumor (Figure S5C); CCND1 amplification is particularly common in ER-positive tumors and is associated with reduced survival in these patients [39,40,41,42,43].

The IGRN path TF-target: HDAC2/EZH2-CDKN1A was activated in the interplay between T cells and malignant cells in m_cluster5 (Figure 5B). A previous study indicates that HDAC2 could arise as a new potential index of aggressiveness and a therapeutic target against breast cancer [44]. Signaling molecules such as FOXA1, EGR1, and ESR1 were also identified in the T cells’ interaction with m_cluster2 (Figure 5B). In addition, as malignant cells orchestrated by immune cells exhibit distinct transcription factor expression patterns (Figure 5C,D), they potentially exert diverse influences on the functionalities of various malignant cell types, thus exhibiting the heterogeneity of patients and spatial ecological niches.

### 3.7. Combining of Single-Cell RNA Sequencing Discovery with ST Analysis

**Figure 6:**
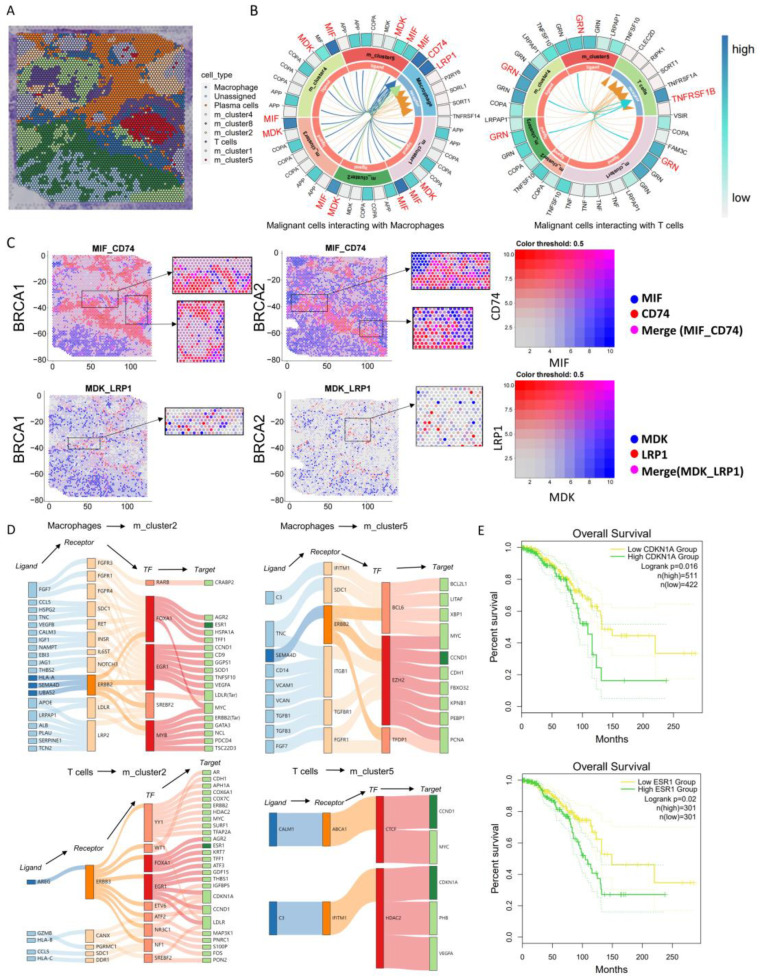
ST data analyzing malignant cell interactions with macrophages and T cells. (**A**) ST data (BRCA1) clustering and definition of cell types. (**B**) Ligand–receptor interactions between malignant cells and macrophages/T cells in ST data (BRCA1). (**C**) Estimates of ligand and receptor pathways in tissue patches in ST data, where blue represents ligand, red represents receptor, and pink represents ligand–receptor co-localization (darker colors have higher expression). (**D**) IGRNs of macrophage/T cells acting on m_cluster2/m_cluster5 constructed from ST data (BRCA1), which includes ligand–receptor pairs, TFs, and their respective targets. (**E**) Kaplan–Meier estimates of overall survival from BRCA patients in TCGA based on targets (CDKN1A, ESR1).

To visually verify the consistency of interaction signaling molecules between immune cells and tumor cells, we utilized annotated ST data (BRCA1) (Figure 6A). In the ST datasets, we found MIF-CD74 and MDK-LRP1 interactions between malignant cells and macrophages, which was true for multiple malignant cell clusters (Figure 6B). These findings underscore the significance of these ligand–receptor interactions in mediating cellular communication within the TME. Furthermore, by examining the spatial ligand–receptor expression patterns in the BRCA1 and BRCA2 tissue samples, we observed the spatial co-expression of the validated ligand–receptor pairs (Figure 6C). This spatial co-expression provides additional evidence of the physical proximity and potential functional interplay between malignant cells and immune cells.

In addition, we constructed IGRNs between invasive frontline malignant cells and macrophages/T cells using the same approach for ST data (BRCA1), and we identified several key TFs that are identical to the previous network, including EZH2, FOXA1, EGR1, and HDAC2, as well as target genes such as CDKN1A, CCND1, and ESR1 (Figure 6D). The CDKN1A gene product is a p53 downstream effector, which participates in cell differentiation, development processes, repair, apoptosis, senescence, migration, and tumorigenesis [45]. Estrogen receptor alpha (ERα) is a nuclear hormone receptor and a key driver of tumorigenesis and tumor progression in these breast cancers, and it is a key treatment target and a biomarker predictive of response to endocrine therapy [46]. To further explore the clinical implications of these findings, we assessed the prognostic features of CDKN1A and ESR1 using GEPIA2. The higher expression levels of CDKN1A and ESR1 in tumors are associated with poor survival outcomes (Figure 6E).

### 3.8. Independent Validation of Main Signaling Molecules in IGRN

**Figure 7:**
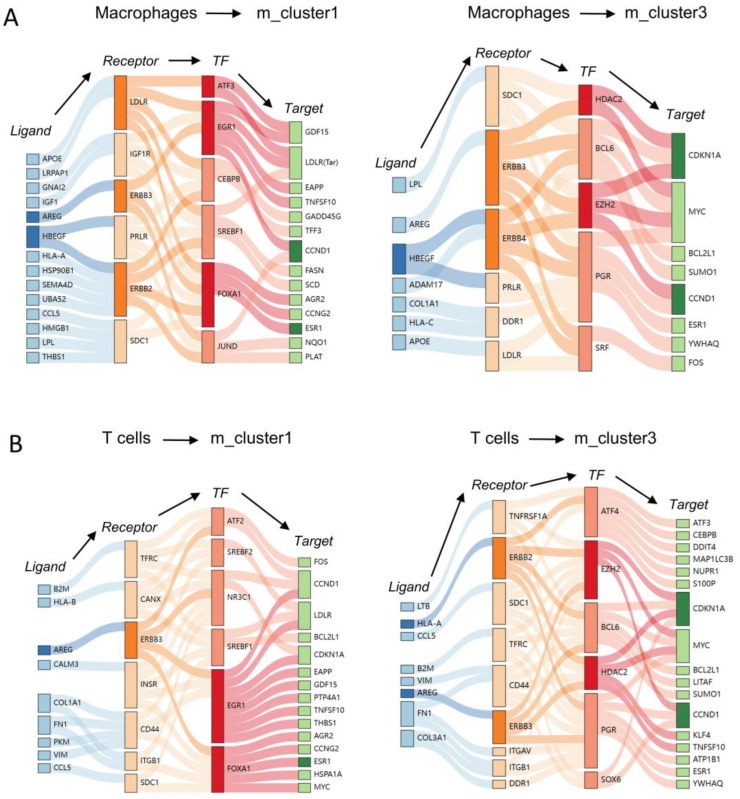
IGRN in independent single-cell validation datasets. (**A**) Macrophages mediate the IGRN of the m_cluster1/m_cluster3 malignant cell population. The network involves ligand–receptor interactions, TFs, and their associated targets. (**B**) T cells mediate the IGRN of the m_cluster1/m_cluster3 malignant cell population. The network involves ligand–receptor interactions, TFs, and their associated targets.

To validate the accuracy of the single-cell and ST workflows in this study, as well as the robustness of the gene regulatory network screening methodology, and to ensure the consistency of the signaling molecules identified, we acquired an independent dataset of ER+ breast cancer single-cell transcriptomes (Figure S6) for validation purposes. Employing a congruent approach, we inferred the principal malignant cell populations situated at the invasive front by integrating scRNA-seq and ST data. Subsequently, we constructed IGRNs utilizing this validation dataset. Through this independent validation, we consistently pinpointed several key TFs within the IGRNs, notably EZH2, FOXA1, EGR1, and HDAC2, along with their target genes such as CDKN1A, CCND1, and ESR1 (Figure 7A,B). These findings mirror our earlier discoveries, suggesting the prevalence of these regulatory relationships among malignant cells and macrophages/T cells. The outcomes from this validation cohort further affirm the viability of our single-cell and ST analysis methods, reinforcing the accuracy of our gene regulatory network construction.

## 4. Discussion

In this study, we performed a comprehensive analysis of the TME at the invasive front using scRNA-seq and ST data in combination with the IGRN, which led to an understanding of the intercellular gene regulatory network (IGRN) at the molecular level and revealed plausible signal transduction relationships. This integrated approach to spatial profiling mitigates the limitations of the individual methods. The results showed that the incorporation of spatial information provided a more intuitive image of the complex TME.

Malignant cells at the invasion front are more invasive and more likely to invade surrounding normal tissue, causing tumor growth and spread. It is therefore particularly important to infer malignant cells at the invasive front. CNVs are widely distributed in the human genome, and the total number of nucleotides they cover greatly exceeds the total number of SNPs, greatly enriching the diversity of genetic variation in the genome [47]. Therefore, CNVs are considered essential features of mutated genomes and are incorporated into a single-cell data analysis to identify malignant and normal cells. By combining CNV to infer malignant cells, as well as the proliferation ability and spatial location information of malignant cells, we inferred the main malignant cell population located at the invasion front.

The immune microenvironment of tumors plays an important role in the occurrence and development of cancer. We found that as the disease progresses, the infiltration of T cells and macrophages in the immune microenvironment increases significantly and is enriched at the tumor invasion front. Through survival analysis, we found that the high infiltration of T cells and macrophages was associated with poor patient survival. Cytotoxic T cells are often associated with immune responses and tumor suppression, while Tregs cells may enhance tumors’ immune evasion capabilities. We further explored and found that in tumor samples, the proportion of Tregs cells increased and was associated with poor patient prognosis, while the decreased proportion of cytotoxic T cells was also associated with poor patient prognosis (Figure S7). This suggests possible immune evasion and immune suppression in tumors. In addition, the signaling network of the tumor immune microenvironment involves a variety of cell signaling pathways and molecular regulatory mechanisms, affecting the proliferation, metastasis, and invasion of tumor cells [48,49]. Our analysis reveals that in ER+ breast cancer, malignant cells mediate the generation of an immunosuppressive microenvironment in macrophages through the MIF-CD74 and MDK-LRP1 ligand–receptor axis. In short, the formation of a tumor immunosuppressive microenvironment leads to the continued proliferation of malignant cells.

At present, most of the cell–cell communication is ligand–receptor based. Our study provides a bioinformatics pipeline for constructing an intercellular gene regulatory network. The construction of an IGRN further elaborates the process of downstream gene regulatory signal transmission after cell–cell communication and improves the integrity of the cell–cell communication and cellular gene regulatory network [6]. By establishing IGRNs between aggressive frontier malignant cells, T cells, and macrophages, key regulatory relationships such as FOXA1-ESR1 and EZH2-CDKN1A have been discovered. In ER+ breast cancer, ESR1 mutations have become a key mechanism of resistance to endocrine therapy [50]; therefore, CDKN1A becomes a very promising target for cancer treatment [51]. This regulatory relationship promotes the further invasion and proliferation of tumors and reduces the survival time of patients. A validation set of the regulatory relationships between the above-mentioned regulatory molecules yielded similar results. This ensures the validity of the IGRN as well as the consistency and reliability of the analyses, deepening the understanding of the cellular mechanisms of macrophage/T cell-mediated malignant cells at the frontiers of ER+ breast cancer invasion, as well as informing the screening of patients for prognostic and therapeutic markers.

ScRNA-seq data provide a high-resolution view of individual cells, allowing us to capture changes in cell populations across cell types and states. Further, ST data compensate for the lack of spatial distribution information in single-cell data. In addition, the construction of gene regulatory networks can help us understand the complexity of intercellular interactions and gene regulation more deeply. However, cell–cell interaction and regulation are mostly studied in regular TMEs, while it is the most important to study them in the tumor invasive boundary context [52], since they are related to tumor invasion and metastasis, which directly affects cancer progression and prognosis. Such research involves careful curation and the combination of ST data and single-cell sequencing data. In our study, we demonstrated that the construction of IGRNs by combining scRNA-seq and ST data allows us to correlate gene expression with cellular location, which contributes to a more comprehensive and in-depth illustration of the complexity of gene regulation inside and outside the cell.

Overall, this study elucidates the crosstalk between immune cells and malignant cells at the forefront of ER+ breast cancer invasion by combining scRNA-seq and ST data with an IGRN. With the fast development of spatial multiomics technology, and the fast growth of single-cell multiomics data, in the future, the combination of single-cell and spatial multiomics data with the analysis of intercellular regulatory networks will reveal the regulatory mechanisms of complex biological systems and help us to identify key pathways, regulatory factors, and potential therapeutic targets, which will provide important guidance for disease research and drug development.

## Data Availability

The scRNA-seq dataset provided in this study can be obtained through the GEO series accession numbers GSE176078, GSE161529, and GSE164898, and the ST dataset can be obtained through the 10x genomic (Pleasanton, CA, USA) database, The ST data for BRCA1 were downloaded from https://www.10xgenomics.com/resources/datasets/human-breast-cancer-visium-fresh-frozen-whole-transcriptome-1-standard, accessed on 9 September 2022. The ST data for BRCA2 were downloaded from https://www.10xgenomics.com/resources/datasets/invasive-ductal-carcinoma-stained-with-fluorescent-cd-3-antibody-1-standard-1-2-0, accessed on 9 September 2022. The TCGA data were downloaded from https://xenabrowser.net/datapages/?cohort=TCGA%20Breast%20Cancer%20(BRCA)&removeHub=https%3A%2F%2Fxena.treehouse.gi.ucsc.edu%3A443, accessed on 2 November 2022.

## Supplementary Materials

The following supporting information can be downloaded at: https://www.mdpi.com/article/10.3390/genes15010100/s1, Figure S1. Spatial location information of cell populations demonstrated by ST data. (A) This figure shows the distribution of tissue-derived and patient-derived cells in the scRNA-seq data. (B) Seurat analysis of cell population spatial distribution. (C) CellTrek evaluation of cell population spatial distribution. (D) Box plot demonstrating the intercellular distance of epithelial cells from major immune cells and stromal cells assessed using CellTrek. Figure S2. Copy number changes in epithelial cells, tumor gene expression, and intercellular correlations. (A) Copy number profiles for luminal cells, cycling cells, and basal cells. (B) ER+ breast tumor malignant cell marker gene expression in luminal cells, cycling cells, and basal cells. (C) Correlation map depicting correlation strength between cell clusters. Figure S3. Malignant cell developmental trajectories, and proportions in patients. (A) Pseudotrajectory of malignancy in three distinct states. (B) Pie chart showing the proportion of cells in the differentiated state of malignant cells. (C) The proportion of malignant cells within the patient’s cell population. Figure S4. Subdivided subpopulations of myeloid cells, and analysis of proportional changes and survival of plasma cells. (A) UMAP representation for myeloid cells, highlighting distinct cellular clusters. (B) Spatial distribution of myeloid cell subpopulations. (C) Cell ratio examination within single-cell data and survival analysis of plasma cells, employing TCGA-BRCA dataset. Figure S5. Intercellular gene regulatory networks (IGRNs), differential gene expression. (A) IGRNs of macrophage-m_cluster3. (B) IGRNs of T-cell-m_cluster3. (C) Differential genes between tumor samples and normal samples. Figure S6. Independent validation on single-cell set cell mapping. (A) UMAP plots effectively display cell type clusters within the single-cell independent validation set of high-quality cells. (B) A single-cell atlas of epithelial cells. (C) A heatmap illustrates the extensive copy number variation (CNV) profile across different ductal cell clusters. Notably, red and blue hues denote elevated and reduced CNV levels, respectively, with normal epithelial cells serving as reference cells. (D) Box plot showing copy number scores of epithelial cell populations. (E) Differentiation trajectories of malignant cells (including time, cell_type and State). (F) Pie chart showing the proportion of cells in the differentiated state of malignant cells. (G) Spatial representation of malignant cell cluster locations within the tissue image. Figure S7. Analysis of proportional changes and survival of cytotoxic T and Tregs cells. (A) Comparative statistical assessment of cytotoxic T and Tregs proportions within cells sourced from normal and tumor samples, based on TCGA data (“****” means *p*-value < 0.0001). (B) Survival analysis utilizing TCGA-BRCA dataset, focusing on cytotoxic T and Tregs cells. Table S1. Clinical data from normal breast and ER+ breast cancer patients.
