# Supplementary figures and images for: Deciphering the Immune Microenvironment at the Forefront of Tumor Aggressiveness by Constructing a Regulatory Network with Single-Cell and Spatial Transcriptomic Data
Source: Genes (Basel). 2024 Jan 15;15(1):100. doi: 10.3390/genes15010100 (PMC10815467; doi:10.3390/genes15010100)

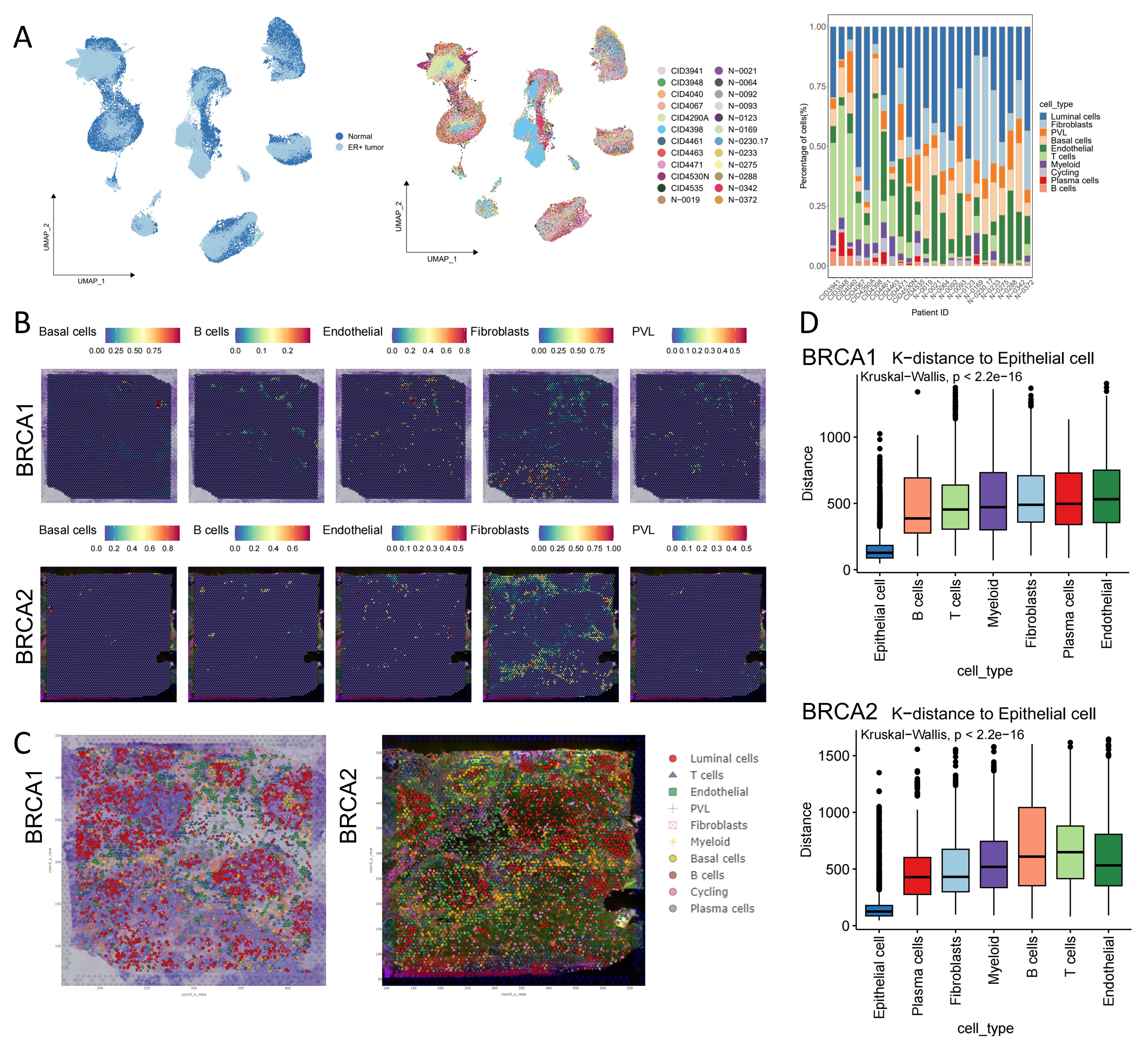

Supplement: Supplementary file 1 [file genes-15-00100-s001.zip › FigureS1.jpg]

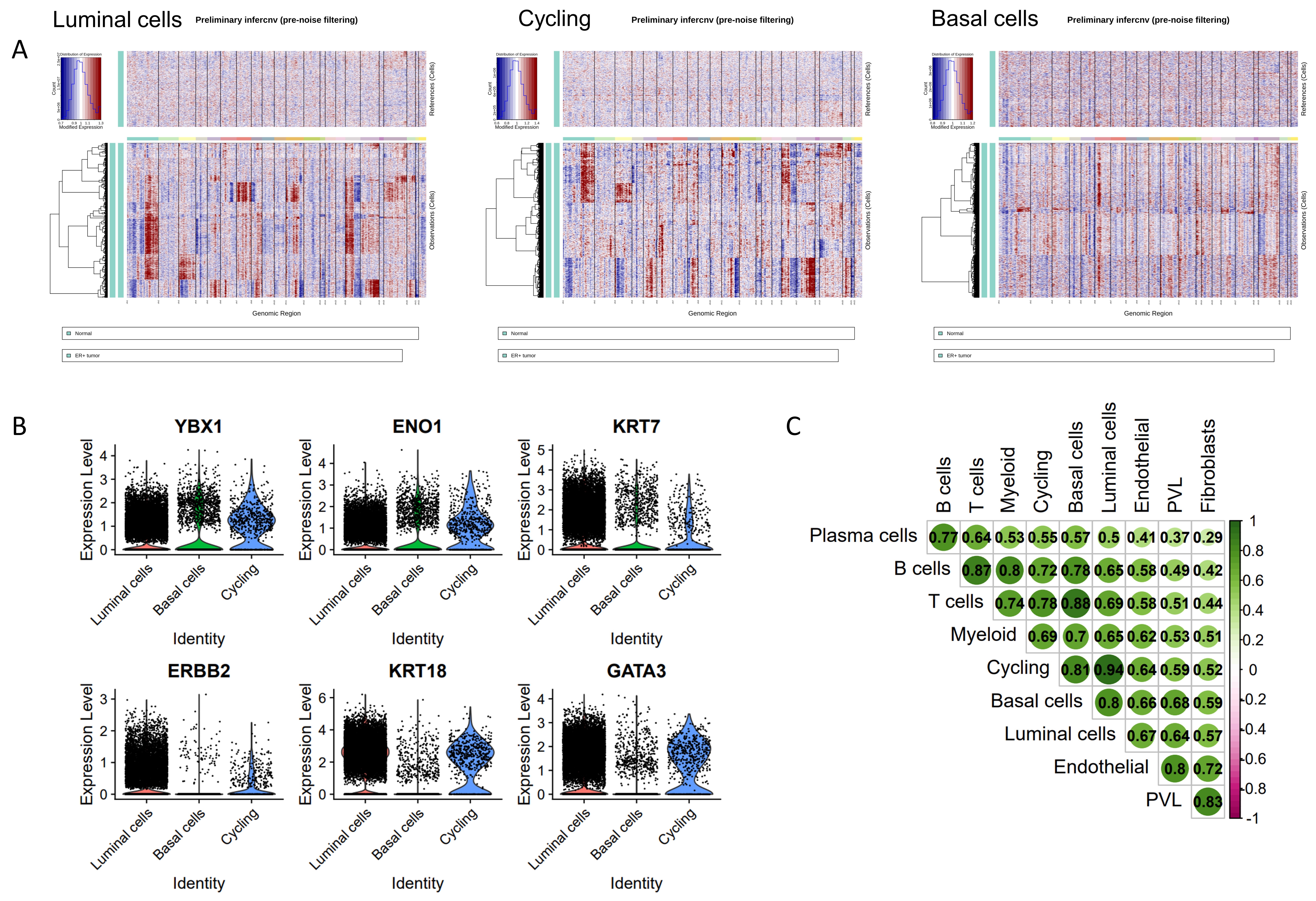

Supplement: Supplementary file 1 [file genes-15-00100-s001.zip › FigureS2.jpg]

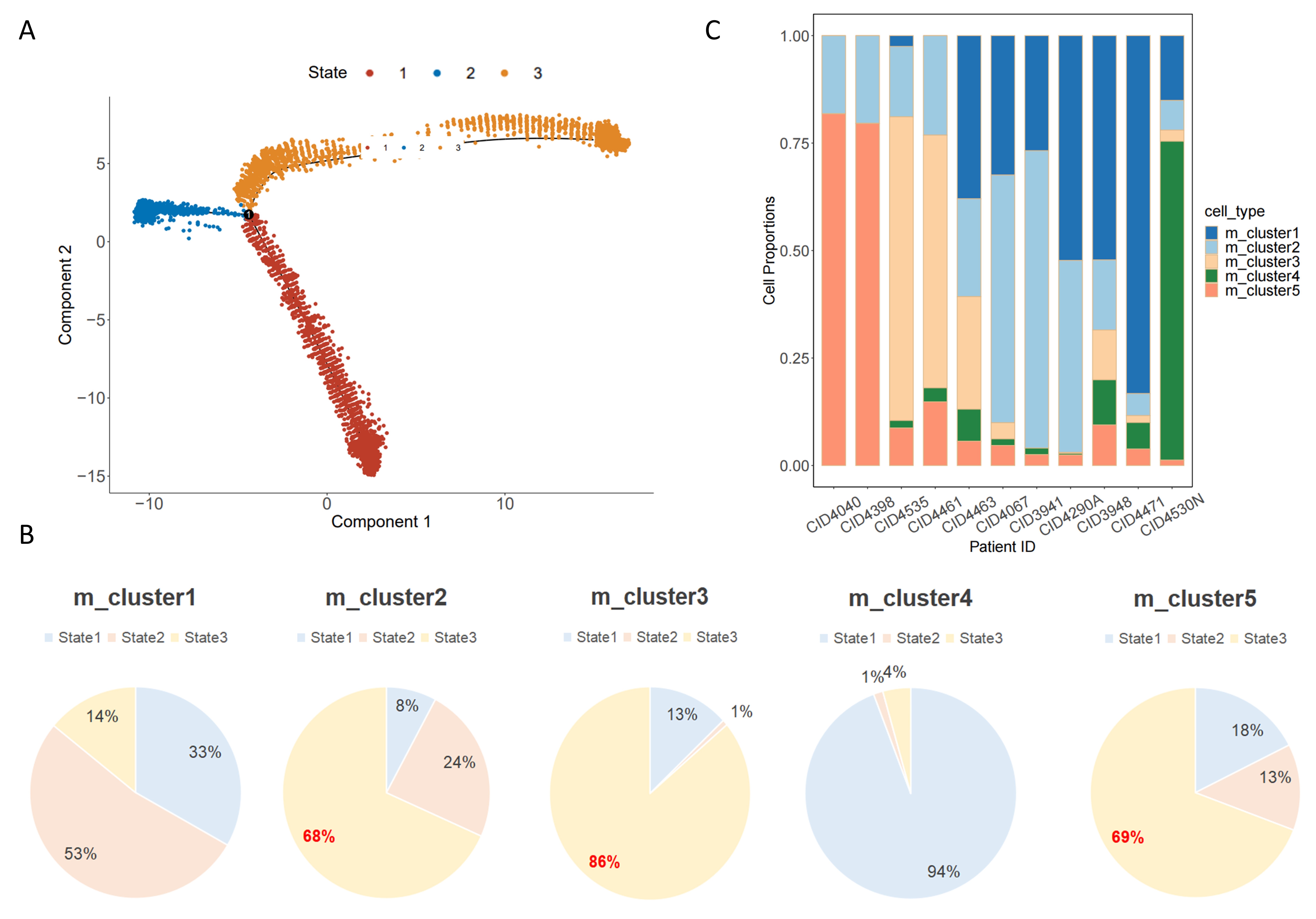

Supplement: Supplementary file 1 [file genes-15-00100-s001.zip › FigureS3.jpg]

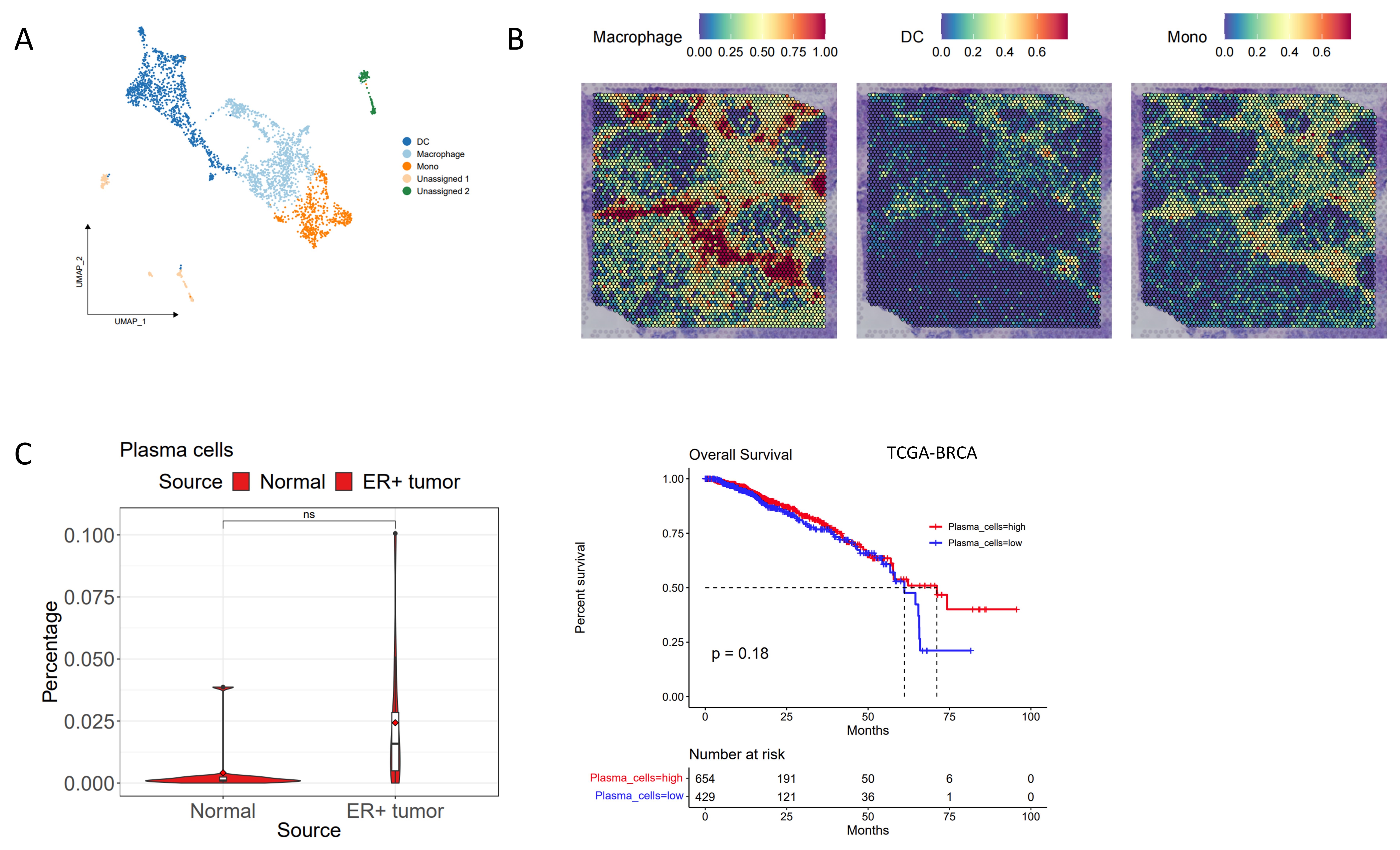

Supplement: Supplementary file 1 [file genes-15-00100-s001.zip › FigureS4.jpg]

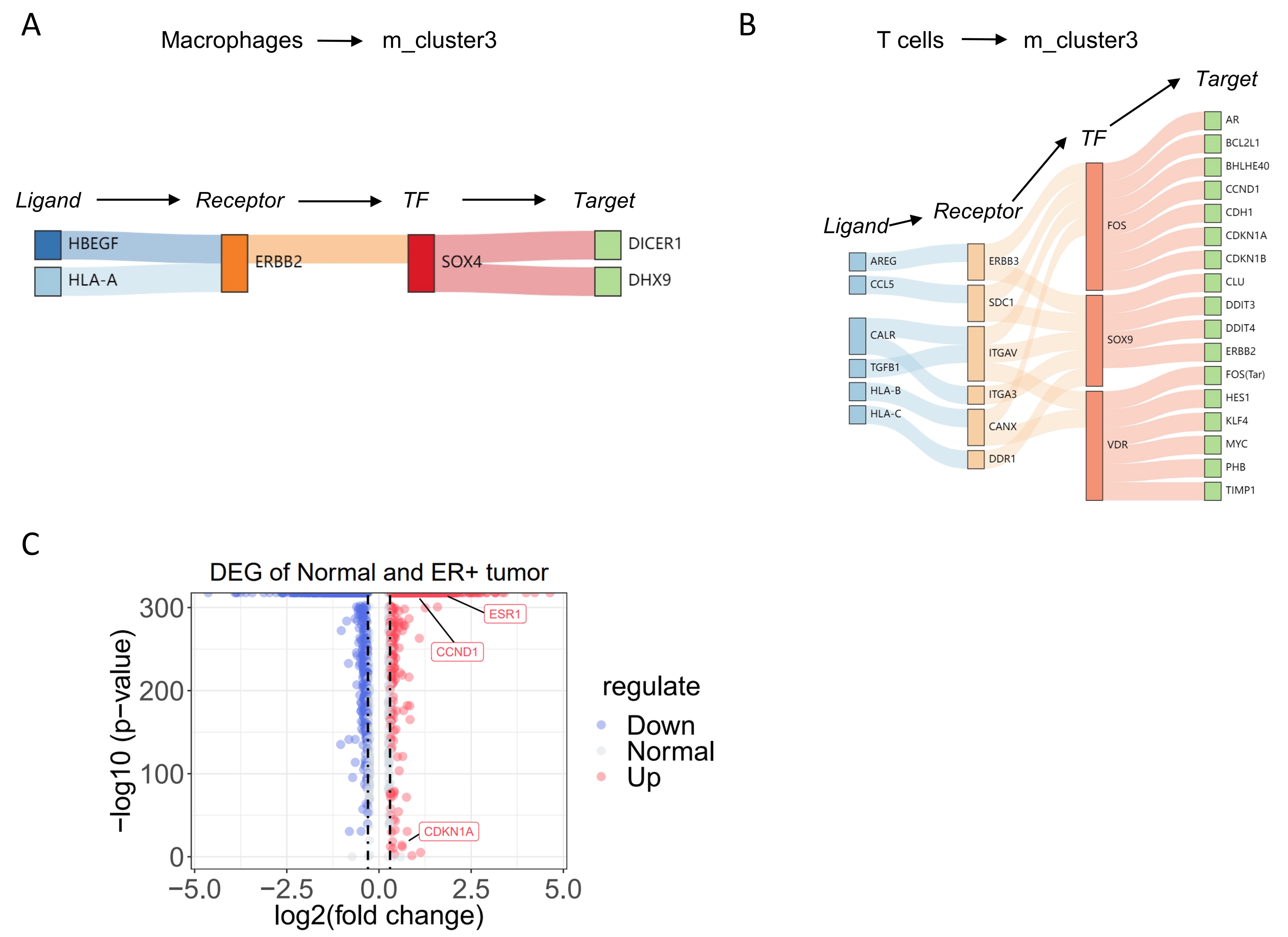

Supplement: Supplementary file 1 [file genes-15-00100-s001.zip › FigureS5.jpg]

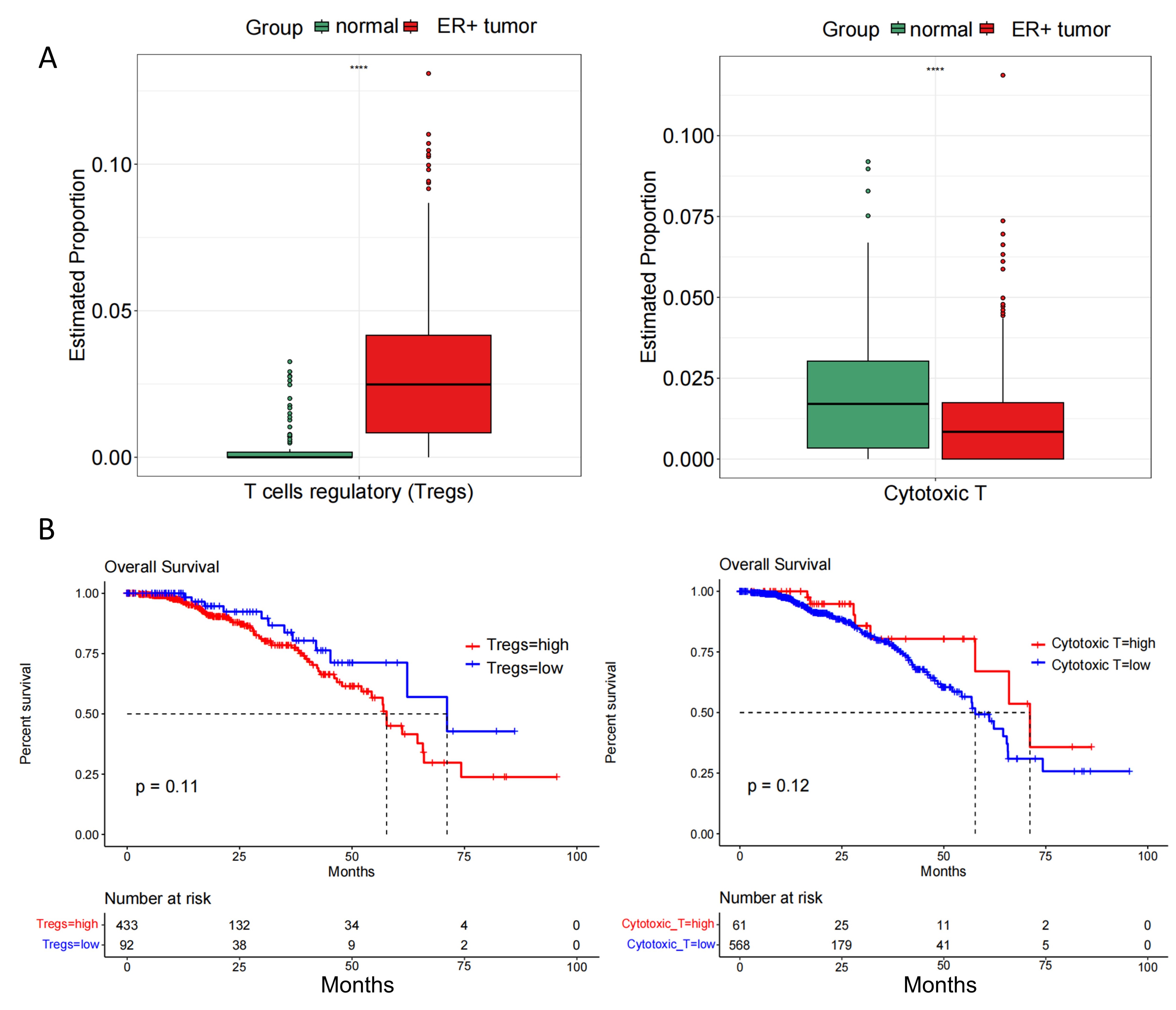

Supplement: Supplementary file 1 [file genes-15-00100-s001.zip › FigureS7.jpg]
